# Spontaneous electrical low-frequency oscillations: a possible role in *Hydra* and all living systems

**DOI:** 10.1098/rstb.2019.0763

**Published:** 2021-03-15

**Authors:** Alison Hanson

**Affiliations:** ^1^ Neurotechnology Center, Department of Biological Sciences, Columbia University, New York, NY, USA; ^2^ Department of Psychiatry, New York State Psychiatric Institute, Columbia University, New York, NY, USA

**Keywords:** *Hydra*, spontaneous electrical low-frequency oscillations, default mode network, integration, self, organism organizer

## Abstract

As one of the first model systems in biology, the basal metazoan *Hydra* has been revealing fundamental features of living systems since it was first discovered by Antonie van Leeuwenhoek in the early eighteenth century. While it has become well-established within cell and developmental biology, this tiny freshwater polyp is only now being re-introduced to modern neuroscience where it has already produced a curious finding: the presence of low-frequency spontaneous neural oscillations at the same frequency as those found in the default mode network in the human brain. Surprisingly, increasing evidence suggests such spontaneous electrical low-frequency oscillations (SELFOs) are found across the wide diversity of life on Earth, from bacteria to humans. This paper reviews the evidence for SELFOs in diverse phyla, beginning with the importance of their discovery in *Hydra*, and hypothesizes a potential role as electrical organism organizers, which supports a growing literature on the role of bioelectricity as a ‘template’ for developmental memory in organism regeneration.

This article is part of the theme issue ‘Basal cognition: conceptual tools and the view from the single cell’.

## 1. Introduction

*Hydra*, the small freshwater cnidarian polyp, has served as a fruitful model organism for numerous cell and developmental biological studies since its discovery over 300 years ago. Recently, *Hydra* has been revived as a model system in neuroscience, where its seemingly ‘simple’ nerve net is not only illuminating the activity of behaviour-generating neuronal circuits with dramatic whole-body *in vivo* imaging [1], but has also revealed an intriguing phenomenon [1]: spontaneous electrical low-frequency oscillations (SELFOs) at the same frequency as those found in the default mode network (DMN) in human brains. Here, the term ‘SELFO’ will be used to refer to organism-wide oscillatory electrical activity of low frequency (typically 0.01–0.1 Hz, but the exact frequency is organism-dependent) that is spontaneously produced independent of external stimuli and does not appear to directly generate behaviour. Such mysterious SELFOs were first observed in *Hydra* and other Cnidaria in the 1960s [2,3], but they were not pursued so their function was not deciphered. These SELFOs were rediscovered in the more recent *Hydra* work, but were similarly set aside in pursuit of behaviour-generating networks, so their function remains unknown [1]. In the 1990s, SELFOs were unexpectedly detected in the human brain with the discovery of the DMN, which has become widely studied and hypothesized to play a role in ‘resting-state’ mental processes, such as spontaneous thought, episodic memory, mind-wandering and self-related processing [4].

Surprisingly, increasing evidence suggests SELFOs are found throughout the living world—including in non-neuronal organisms such as plants [5,6], fungi [7–9], protozoa [10] and bacteria [11–13]—which may point to a fundamental biological function that evolved early in the development of life on Earth. The claim defended here is that SELFOs may have a potential role as *electrical organism organizers*, serving as system-wide integrators and communicators, making them critical for the construction and maintenance of organism unity and coherent, adaptive behaviour. Such a view is consistent with recent suggestions that bioelectrical phenomena may act as a template for developmental memory, including in regeneration, for which *Hydra* has long served as a model organism [14].

The paper is structured as follows. Section 2 presents an overview of *Hydra* as a model system in cell and developmental biology, focusing on what this early work taught us about how multicellular organisms build their bodies. Section 3 addresses early research into cnidarian neurophysiology and behaviour beginning in the 1870s and culminating in the 1960s, which revealed ubiquitous spontaneous neural activity [2,3]. Section 4 introduces the as-yet poorly understood spontaneous electrical activity in human brains, notably the ‘DMN’, its unexpected discovery, and hypotheses concerning its function [4]. Section 5 explores the evidence for SELFOs in other widely divergent organisms. Section 6 advances a highly preliminary hypothesis about what role SELFOs might be playing in biological systems—as organizers of organism construction and persistence—and how *Hydra* is an ideal model system to begin rigorously testing this idea and others that ramify from it.

## 2. *Hydra* as an early model system

The Dutch microbiological pioneer Antonie van Leeuwenhoek discovered *Hydra* in 1702. In his letter to the Royal Society of London, he described finding a number of ‘animalcula’ attached to the roots of ‘green weeds’ he had pulled out of a river in what was then called the Low Countries [15]. These particular ‘animalcula’ appeared to contract and elongate, produce ‘young animalcula’ from their sides, and draw small ‘wheels’ in and out of their bodies. Van Leeuwenhoek included drawings of these organisms along with his letter, but that was the extent of his investigation.

Nearly 40 years later, a Dutch tutor, Abraham Trembley, unaware of van Leeuwenhoek's earlier discovery, collected specimens from a nearby pond and rediscovered a small green polyp. Unlike van Leeuwenhoek, who surveyed many organisms, which happened to include *Hydra*, Trembley took a deep dive into *Hydra* biology and became fascinated with determining whether the organism was an animal or a plant. Plants were then known to regenerate, while animals were not. To settle the issue, Trembley cut the polyp in half. To his surprise, the polyp regenerated its entire body, which suggested to him it was a plant. However, the polyp could also move in complicated ways—including capturing prey, feeding itself using its tentacles and doing ‘somersaults’—abilities classically only associated with animals. Following a series of meticulous experiments in which he both observed and recorded the various behaviours of the polyps and the myriad ways they were able to regenerate themselves from fragments of tissue, Trembley concluded this category-defying polyp was, indeed, an animal that could regenerate itself, just like a plant. He published his landmark results in a series of letters to the Royal Society from 1742 to 1746 [16–19] and in a book cataloguing his studies in 1744 [20], which, together, sparked great interest in the phenomenon of animal regeneration and launched the field of *Hydra* biology.

Since Trembley's initial experiments nearly 280 years ago, *Hydra* has served as an extremely useful model organism for studying a wide variety of biological processes, including: ageing, regeneration, pattern formation, and stem cell maintenance and differentiation [21]. One of the major early discoveries in *Hydra* came in 1909 when Ethel Browne, a graduate student working alongside T. H. Morgan and E. B. Wilson, demonstrated that excising a piece of tissue from the sub-tentacle region of one animal and grafting it onto the body column of another induced the formation of a second body axis—a second head—at the implantation site [22]. As had Trembley, Browne carried out a series of careful grafting experiments that showed this novel property of ‘induction’, resulting in a second body axis, was reproducible and specific to tissue in the sub-tentacle region. Fifteen years later, Hans Spemann and Hilde Mangold performed nearly the same experiment and demonstrated the same effect using amphibian embryos [23]. They dubbed this special piece of ‘inducing’ tissue the ‘head organizer’, for which Spemann received the Nobel Prize in 1935. Mangold died before the prize was awarded and Browne's original work in *Hydra* was never acknowledged [24].

Nevertheless, Browne's discoveries in *Hydra* set the agenda for developmental biological research for years to come in which the primary aim was to determine what made the head organizer tissue so special [25]. What was it about that particular tissue that could induce the formation of another body axis and what were the specific ‘inducing factors’? Numerous tools were developed to identify and localize different molecules and cell types, which led to the finding that the head organizer establishes a gradient of molecules across developing organisms, a kind of ‘molecular map’ cells can ‘read’ to ‘know’ what kind of cell to become and their proper location within the organism [26]. Unexpectedly, these ‘molecular maps’ subsequently were found to be highly conserved among multicellular animals. The same molecules (e.g. Wnt, BMP, Hox) appeared to be used in essentially the same way by all organisms, from *Hydra* to humans, to establish body-axis polarity—the anterior–posterior and dorsal–ventral poles—as well as tissue types and overall body plan [27]. This demonstrated that studying fundamental biological phenomena in basal metazoans, like *Hydra*, can illuminate these same processes in more complex animals.

## 3. Neurophysiology and behaviour research in Cnidaria

In parallel to the primarily developmental studies in *Hydra* was a lesser-known line of research focused alternatively on behavioural and neurophysiological studies. This line of work began in the 1870s with George Romanes, one of Charles Darwin's disciples working in England, and Theodor Eimer, a zoologist working independently in Germany [28]. Both became fascinated by the complex behaviours of jellyfish, larger Cnidaria related to *Hydra*, including their ability to move in ‘purposeful’ ways and capture and ingest their prey (as does *Hydra*), which suggested the presence of a nervous system. Unlike their predecessors—including Louis Agassiz and Ernst Haeckel—who focused almost exclusively on identifying the structure of this presumed nervous system using various histological methods, with equivocal and controversial results, Romanes and Eimer aimed to prove these basal metazoans possessed a nervous system by studying its potential function in coordinating the animal's behaviour. They both made significant progress along these lines, which they published one month apart in 1874 [29,30], but both men died prematurely, ending this line of investigation.

Work on the neurophysiology and behaviour of Cnidaria was revived in the 1930s when Carl Pantin became interested in how the nervous system (then known to be in the form of a diffuse nerve net) controlled the muscles of the sea anemone, *Actinia* [28]. Pantin, like Romanes and Eimer, made considerable progress in determining how behaviour is coordinated in this system, which he summarized in his 1952 Croonian Lecture [31], but he, like his predecessors, was limited by the tools of his time. True neurophysiology did not begin in earnest until the 1950s, when the advent of both electron microscopy and electrophysiology enabled more sophisticated studies of the structure and function of cnidarian nervous systems. A major breakthrough in electron microscopy was identification of synapses with dense core vesicles in jellyfish neurons, confirming basal metazoans possess neurons with structures very similar to neurons in more complex organisms, such as mammals [32]. Concurrently, significant advances were being made into the electrical properties of these early nervous systems. A newly developed microelectrode inserted into the extracellular space adjacent to a jellyfish neuron enabled Horridge to record the first action potential in a cnidarian in 1953 [33]. This work inspired others to use microelectrodes to investigate the electrical properties of other cnidarians, including *Hydra* [28].

In the 1960s, Passano & McCullough published a series of papers [34–37] summarizing their experimental work on the electrical activity and behaviour of *Hydra*. Their careful analysis led them to three conclusions. First, *Hydra* exhibits spontaneous, rhythmic behaviour independent of the surrounding environment, although it can be influenced by external circumstances [35,37]. Second, *Hydra* possesses a nervous system composed of two ‘pacemaker systems’ that control specific behaviours [34–37]. Finally, these electrical ‘pacemaker systems’ also exhibit spontaneous, rhythmic activity, some of which was not associated with any behaviour, which they termed ‘cryptic’ [34–36]. These same features were found by other investigators of the time in several other cnidarians, suggesting significant conservation of function among these early animals [2,3]. These findings led Passano to propose a model featuring a ‘hierarchy of pacemakers' in which one pacemaker would serve to coordinate all the others to ensure coherent animal behaviour, giving the surprising appearance of certain ‘central nervous system’ features in these seemingly simple, radially symmetric nerve nets [2,3]. This work substantially contributed to understanding how electrical activity in *Hydra* is related to its behaviour. Nevertheless, given the continuing limitations of the available tools, major questions remained unanswered and the research ground to a halt—until now.

**Figure 1.**
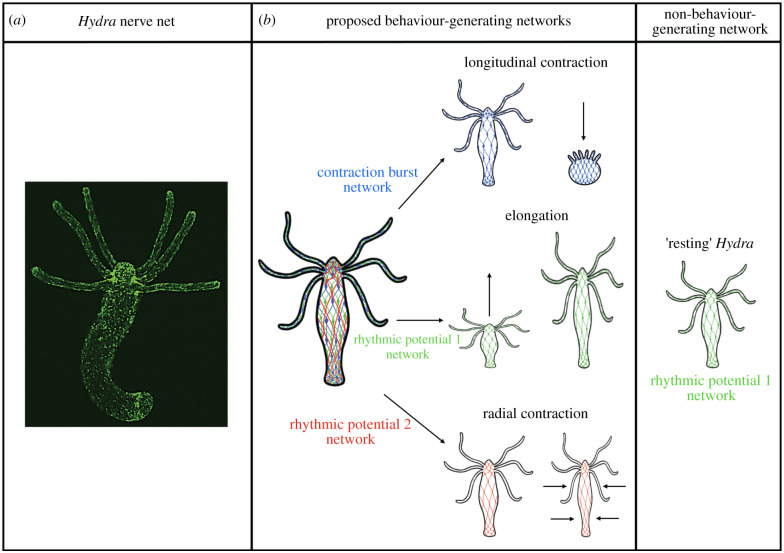
The *Hydra* nerve net and its proposed functions. (*a*) The *Hydra* nerve net is visualized by labelling neurons with GFP and imaging with a spinning disc confocal microscope. (*b*) The *Hydra* nerve net is composed of three major proposed behaviour-generating networks: the contraction burst network correlated with longitudinal contraction, the rhythmic potential 1 (RP1) network correlated with elongation, and the rhythmic potential 2 (RP2) network correlated with radial contraction. In addition to these proposed behaviour-generating networks, the RP1 network is also active during *Hydra* ‘rest’ when the animal is kept under constant external conditions and exhibits no observable behaviour [1,34].

A major goal of modern neuroscientific research is to record the activity of all neurons in a behaving animal at single-neuron resolution to enable visualization of emergent phenomena of the whole system that would otherwise be missed when recording only one neuron at a time, as is the case when using microelectrodes [38]. The development of fluorescent genetically encoded calcium indicators (GECIs) in the early 2000s allowed all-optical imaging of previously inaccessible nervous systems [39]. With its small size, transparent body, and diffuse nerve net lacking any well-defined brain or ganglia (figure 1*a*), *Hydra* has proved to be an ideal model system for optical imaging of all of its neurons at single-cell resolution at the same time [40]. This feat was accomplished in 2017, when the activity of nearly all neurons in *Hydra* were imaged simultaneously using a transgenic animal expressing the GECI GCaMP6s in its neurons [1]. This work revealed two fundamental features of the *Hydra* nervous system that are mostly consistent with Passano & McCullough's earlier work. First, the *Hydra* nervous system is composed of three major neural networks (or ‘ensembles’). Second, it is spontaneously active (see figure 1*b* for details).

The discovery of three major functional ensembles within the *Hydra* nervous system sheds light on a long-standing question in neuroscience: what is the ‘fundamental unit’ of the nervous system? Originally hypothesized to be a continuous ‘reticular meshwork’ functioning as a single unit [41], Ramon y Cajal and Sherrington transformed understanding of the nervous system with the ‘neuron doctrine’—the idea based on Schleiden & Schwann's ‘Cell Theory’ [42] that individual nerve cells are the fundamental units of nervous systems. The neuron doctrine remains neuroscientific orthodoxy, although not without escalating challenge. Today, growing evidence suggests function arises at the level of groups or ensembles of neurons [43,44], somewhere between a continuous meshwork and independent units. That *Hydra*, with one of the earliest nervous systems, a seemingly ‘simple’ nerve net, carved itself into three such functional ensembles, supports this idea. Once again, this basal metazoan appears to be teaching us something fundamental about biology; in this case, neurobiology.

The rediscovered second feature of the *Hydra* nervous system, its spontaneous activity, arguably has the potential to lead to similarly revolutionary changes in neurobiology. Sherrington's still-influential proposal of the nervous system as effectively a ‘reflex organ’ waiting for environmental stimuli to push the organism to behavioural response [45]—the foundational proposition of the input–output view of information processing [46]—cannot account for spontaneous neural activity that seemingly has no effect on behaviour. As we have seen, however, such ‘cryptic’, non-behaviour-inducing spontaneous activity has been recognized in Cnidaria for more than half a century [2,3]. Although speculated to play a role in coordinating animal behaviour at the time, the function of this activity was left to ‘future work’, which was never done. While poorly understood, findings across diverse Cnidaria were essentially the same: endogenously active nervous systems produced rhythmic, low-frequency pulses even in an unchanging environment and even when organisms were at rest. Why? Why would energetically expensive [47] nervous systems be perpetually active in the absence of a stimulus and in the absence of any discernible behaviour? A clue about the potential role of this low-frequency spontaneous neural activity in comparatively simple organisms comes from an unexpected place: the human brain.

## 4. The default mode network: low-frequency neural oscillations in humans

**Figure 2.**
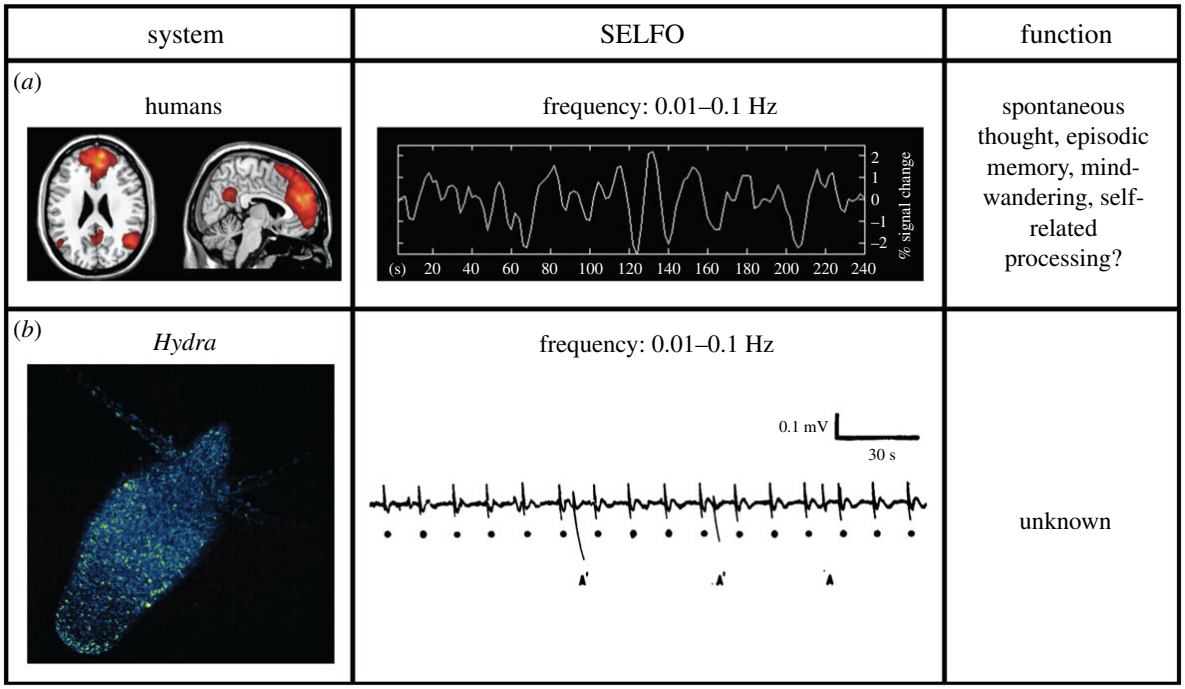
SELFOs in humans and *Hydra*. (*a*) Spontaneous electrical activity in the human ‘DMN’ in a representative subject ‘at rest’ as measured by functional magnetic resonance imaging (fMRI) (left) with its associated time course showing low-frequency oscillations (middle), which are proposed to play a role in the functions listed (right). Left and middle panels adapted from fig. 1a in [51] (copyright 2008 National Academy of Sciences, USA). (*b*) Spontaneous electrical activity in the *Hydra* RP1 network as visualized in *Hydra* ‘at rest’ expressing GCaMP6s in its neurons (left) with a representative time course measured in earlier work with extracellular electrodes showing its low-frequency oscillations (middle) of unknown function. A and A′ indicate asymmetrical epidermal muscle contractions correlated with an electrical potential distinct from rhythmic potential (RP). RP pulses are denoted by black dots and resulted in no observable behaviour. Middle panel adapted from Fig. 1 in [34].

Even before the demonstration of spontaneous activity in cnidarian nervous systems, Hans Berger used his newly invented electroencephalogram to discover spontaneous, rhythmic electrical activity in human brains in 1929, which he termed ‘alpha waves’ [48]. Despite Berger's intriguing early findings, spontaneous brain activity was mostly ignored in favour of the prevailing view of the brain as an input–output machine, active only in response to external stimuli [45]. Almost seven decades passed before neuroscientists Shulman and Raichle independently noticed a paradoxical result while performing human neuroimaging studies designed to detect ‘task-evoked’ activity. A specific brain network appeared to be specifically *inhibited* during tasks and *more active* while subjects were ‘at rest’ with their eyes closed [49,50]. A series of studies verified the presence of intrinsic brain activity in the absence of changing external conditions or goal-directed behaviour and forced Shulman and Raichle to conclude the conventional belief—that only external stimuli generate brain activity—is seriously flawed. This spontaneous, resting-state network became known as the brain's ‘DMN’ (figure 2*a*), which quickly became an area of intense investigation [4].

While much work has been done on the human DMN since its discovery 20 years ago, its function remains debated. It is believed to be involved primarily in ‘resting-state’ mental processes, such as spontaneous thought, episodic memory, mind-wandering, and self-related processing [4]. Numerous studies have shown significant overlap between resting-state neural activity in cortical midline structures thought to compose the DMN and those active during self-related processing [52,53]. These findings have been replicated using a variety of self-specific versus non-self-specific stimuli in multiple domains, including facial, emotional, verbal, spatial, motor and memory, in which subjects routinely respond more robustly to self-specific versus non-self-specific stimuli [52,53]. In each domain studied, the same cortical midline structures active in the DMN at rest were also activated during self-specific stimulus processing during testing, leading Northoff to propose the DMN might contain, or encode, self-specific information [54]. In addition to these findings, mounting evidence shows disruption of DMN activity via psychedelics or meditation correlates with ‘ego dissolution’, or the loss of a sense of self, consistent with the idea that the DMN might play an important role in the formation of the self in humans [55–60]. However, precisely what the self is and how the DMN might contribute to it, remains obscure.

A clue to how the DMN might contribute to the human self may come from what has been learned about spontaneous brain activity in general in recent years. We now know the human brain produces numerous spontaneous neural oscillations spanning a wide range of frequencies from ultraslow (0.01–1.0 Hz) to ultrafast (200–600 Hz) [61,62]. The same neural oscillation frequency distribution found in humans has been identified in all mammalian brains studied to date; such a robust frequency structure is one of the most highly conserved features of mammalian brains [61]. We also know intrinsic brain activity consumes up to 20% of total body energy, so it cannot be mere ‘noise’, as had been assumed for most of the twentieth century [63]. These two facts—a highly conserved structure and a high energetic cost—suggest that spontaneous brain activity is likely critical for brain function [61,63], although for what is unclear. One proposal, by Buzsáki, envisages a hierarchy of integrating oscillators that form the functional or ‘syntactical’ units of the elusive ‘neural code’ where faster, smaller and more local oscillations become entrained, integrated, or ‘read’ by slower, larger and more global oscillations [64]. The highest-frequency neural oscillations function as the ‘letters’ of the code, which are integrated or ‘read’ by lower-frequency oscillations that form ‘words’, which are integrated or ‘read’ into ‘sentences’ by the next lowest-frequency level, and so on. Although Buzsáki does not explicitly say so, the theory implicitly assumes the presence of an ultimate downstream integrator at the lowest frequency level, which ‘reads’ all the higher-frequency information. Interestingly, the DMN has been found to oscillate in such an ultraslow range (0.01–0.1 Hz) (figure 2*a*) [51,65–67], making it a potential candidate for an ultimate brain integrator.

In addition to its frequency, the structure of the DMN may also provide clues to its function. The overall ‘small-world’ network architecture of the brain is composed of many short, local connections and few long-range connections between nodes. In this picture, the DMN appears to serve as one of the brain's main integrators that connects major connection-rich ‘rich hubs’ via long-range, thickly myelinated axons [68–71]. This network architecture puts the DMN in a central position in the brain (figure 2*a*), in which it both receives and sends information rapidly among otherwise segregated local brain regions. It is believed the DMN receives exteroceptive input from all of the primary sensory areas as well as interoceptive input from the insula, thalamus, hypothalamus, midbrain and brainstem, and, in turn, can rapidly send information back to and between these same areas [4,52,72,73]. Thus, in addition to oscillating at the lowest frequency in the brain, the DMN seems to also be in a unique structural position to act as the ultimate downstream integrator, as implicitly predicted by Buzsáki's theory [64].

Another way to think about the potential role of the DMN in human self-construction is as the top layer of the hierarchical predictive coding ‘self-model’ as put forth by Friston [74,75]. Like Buszáki's theory, which predicts the need for an ultimate brain integrator or ‘reader’ (i.e. a ‘self’), a hierarchical predictive coding model also implies the need for an ultimate brain integrator or ‘predictor’ (also a ‘self’) at the top of the hierarchy. According to predictive coding brain models, prediction error is passed up the hierarchy from the low-level primary, unimodal sensory areas to the ultimate, multi-modal ‘predictor’ at the top of the hierarchy, which contains a high-level abstract representation (of the ‘self’) that then passes predictions back down to the lower levels [74,75]. In this way, the DMN, oscillating at the lowest frequency in the brain, might act as the brain's ultimate information integrator, receiving input from all the lower-level, otherwise isolated units (oscillating at higher frequencies), and passing on one unified ‘self’ prediction back down to generate coherent, adaptive behaviour (figure 4*c*).

Together, these findings suggest the DMN may be implementing a top-down control mechanism in the human brain as it receives bottom-up information from all brain areas (which oscillate at higher frequencies) and may, in turn, constrain these lower levels via its slow-wave oscillations, while also rapidly communicating its unified output to all brain regions via its synchronous electrical activity to maintain organism unity (i.e. a coherent ‘self’). Hence, the human ‘self’ may be constructed bottom-up with the DMN emerging as the ultimate neural integrator and top-down ‘enslaver’ of all the lower levels of organization in the brain. Importantly, this view of the human self does not imply the DMN *is* the self or that the self is a *thing* located *in* the DMN. Rather, it suggests the self is an ongoing *process* in which the DMN continuously receives internal and external sensory information and adaptively updates its predictive model of itself and the world. Although evidence is accumulating connecting the DMN to the human self, its precise function and mechanism remain unclear and the speculative hypotheses put forth here remain untested owing to the difficulties of both imaging and manipulating human brain activity.

## 5. SELFOs in the living world

The presence of SELFOs in cnidarians and humans (figure 2) raises a question: are they found elsewhere? As already mentioned, the same cortical oscillation frequency distribution found in human brains has been found in the cortex of all mammalian brains studied thus far, including in chimpanzees, macaques, sheep, baboons, pigs, dogs, cats, rabbits, guinea pigs, rats, hamsters, gerbils, mice and bats [61]. In addition to the low-frequency cortical DMN, there is evidence for subcortical DMN nodes in the midbrain and brainstem that are highly conserved among mammals and co-active with cortical DMN nodes, thus forming a cortical–subcortical DMN [72,73,76]. Using new functional magnetic resonance imaging (fMRI) techniques, SELFOs have recently been observed in human, non-human primate, and rat spinal cords, indicating these oscillations pervade the entire mammalian central nervous system [77–80].

Spontaneous neural activity is not specific to mammals, however. Zebrafish brains generate a wide range of spontaneous oscillation frequencies, including the ultraslow-frequency range (0.01–0.1 Hz) [81], although their function remains mostly unknown. Brain-wide oscillations of a variety of frequencies have also been recorded in a wide range of insects, including moths [82], locusts [83], water beetles [84], honeybees [85] and flies [86]. Although most of this work has been focused on stimulus-evoked activity and higher-frequency oscillations, an ultraslow-frequency (0.01–0.1 Hz) spontaneous network has been identified in flies [87], the function of which remains to be determined. At the base of the metazoan lineage sit Cnidaria, which possess the earliest known nervous systems: radially symmetric nerve nets that appear to universally generate SELFOs of unknown function (figure 2*b*) [2,3]. Thus, the evidence points to the presence of SELFOs not only in all mammals, but in all animals with a nervous system, despite substantial differences in size and structure.

**Figure 3.**
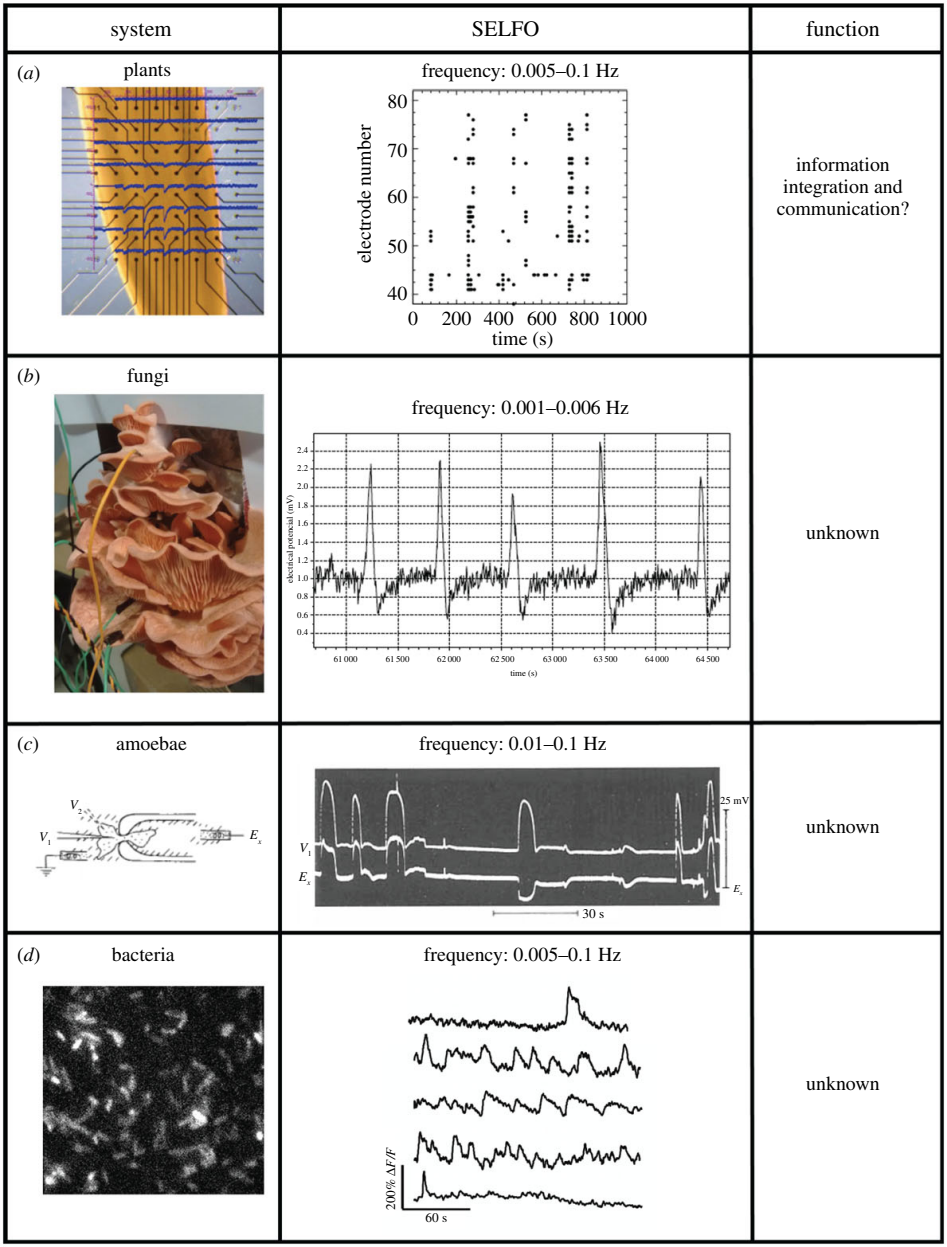
SELFOs in organisms without nervous systems. (*a*) Electrophysiology of plants (*Zea mays*) was investigated using a multi-electrode array in plant roots (left). An example electrical recording shows SELFOs (middle), the function of which is unknown, but a role in information integration and communication has been proposed [6,88]. Figure adapted from Figs 1d and 2 in [5]. (*b*) Electrophysiology of fungi (*Pleurotus djamor*) was investigated using extracellular electrodes placed in the cap and stalk of fruit bodies (left). An example electrical recording shows SELFOs (middle) of unknown function. Figure adapted from Figs 1b and 3b in [7] (http://creativecommons.org/licenses/by/4.0/). (*c*) Electrophysiology of single amoebae (*Chaos chaos*) was investigated using both intra- (*V*_1_) and extra- (*E_x_*) cellular electrodes while the amoeba was held stationary in a glass chamber (left). An example electrical recording shows SELFOs (middle) of unknown function. Figure adapted with permission from Fig. 4a in [10]. (*d*) Electrophysiology of single bacteria (*Escherichia coli*) was investigated using the fluorescent genetically encoded voltage indicator PROPS (proteorhodopsin optical proton sensor) (left). Fluorescence intensity of individual bacteria over time shows spontaneous low-frequency oscillations in membrane potential (middle) of unknown function. Figure adapted with permission from Movie S1 and Fig. 2a in [11].

What about organisms without neuronal wiring? Do they produce similar electrical activity? The answer is resoundingly affirmative (figure 3). Plants have been known to produce neuron-like action potentials for years [89]. However, recent work using new tools (GCaMP3s) in *Arabidopsis* made this even clearer when calcium-mediated action potentials were observed in response to wounding, which travelled throughout the plant and induced expression of downstream wound-response genes at distant sites [90]. In addition to stimulus-evoked electrical activity, plants also exhibit ongoing SELFOs in the transition zones of their roots (figure 3*a*), the proposed information ‘integration centre’ for the whole plant [5,6,88]. Accumulating evidence suggests the plant root transition zone may serve as a sensory information integrator and coordinator of motor responses in distant stems and leaves in response to changing conditions (e.g. light, temperature, salt stress or wounding) [6,88]. What role SELFOs might play in this process remains to be determined. Similarly, several multicellular fungi have been found to exhibit spontaneous, low-frequency action potential-like spikes [7–9]. The first low-frequency spontaneous ‘action potentials’ were identified in the mature hyphae of the fungus *Neurospora crassa* in 1976 using intracellular electrodes—potentials that were conducted organism-wide and had no clear function [8]. Twenty years later, SELFOs were demonstrated in the hyphae of *Pleurotus ostreatus* and *Armillaria bulbosa*, the frequency of which increased in the presence of various stimuli (e.g. sulfuric acid, water, malt extract and wood) and decreased when the wood stimulus was removed, leading the authors to speculate such SELFOs may be used for organism-wide communication in response to changing external conditions [9]. More recently, a 2018 study using extracellular electrodes placed in the cap and stalk of the oyster mushroom (*Pleurotus djamor*) fruit body also revealed SELFOs with no obvious function (figure 3*b*), although a potential role in organism-wide communication was again proposed [7].

But it does not end there. Spontaneous low-frequency electrical activity has also been observed in unicellular eukaryotic and prokaryotic organisms. In 1964 researchers conducted electrophysiological experiments using both intra- and extracellular electrodes in two freshwater amoebae (*Chaos chaos* and *Amoeba proteus*) in an effort to determine why their cytoplasmic potassium concentrations were so high. Surprisingly, they discovered spontaneous action potential-like ‘spike potentials’ of low frequency (figure 3*c*), which prompted them to study these unexpected phenomena instead. They found the spontaneous ‘spike potentials’ could be modulated by various chemicals (e.g. ethyl ether, cocaine, potassium oxalate, CaCl_2_), but had no discernible effect on the cell's behaviour or morphology, leaving their function obscure [10]. Despite possessing many ion channels [91], the electrophysiology of bacteria was mostly unknown until recently owing to the difficulty of using traditional microelectrodes in very small cells with cell walls. The creation of a fluorescent voltage-sensitive protein in 2011, however, allowed visualization of the dynamic electrical properties of bacteria for the first time, revealing spontaneous, low-frequency action potential-like spikes in *Escherichia coli* not clearly related to behaviour (figure 3*d*) [11]. More recently, *Bacillus subtilis* in biofilms have been shown to engage in long-range electrical signalling via propagation of synchronized low-frequency potassium waves both within and between biofilms to coordinate nutrient sharing [12,13], further suggesting a potential role for low-frequency electrical oscillations in ‘organism’-wide information integration and communication.

Although very little is known about their function, SELFOs of some sort appear to be present in most organisms studied thus far, suggesting an important role in living systems.

## 6. Hypothesis: SELFOs as electrical organism organizers

So far we have reviewed the early discovery of the molecular head organizer in *Hydra*, seen that SELFOs of unknown function exist in the earliest nervous systems, learned how the SELFO in the human brain, the DMN, may contribute to the human self by acting as a brain-wide integrator and communicator, and discovered the widespread presence of SELFOs in other highly divergent phyla. Here, I will attempt to weave these threads together and briefly conjecture that SELFOs may be the ultimate organism-wide electrical information integrators and communicators in all biological systems at all levels of scale, making them critical for maintenance of organism unity and coherent, adaptive behaviour.

Since the discovery of the molecular head organizer in *Hydra* over 100 years ago, much has been learned about how organisms build their bodies [26,27]. That is, we have learned much about the *spatial* domain of biology—how multiple independent units (e.g. proteins in cells and cells in multicellular organisms) are coordinated in space to form a unified, structural whole. However, much less is known about the *temporal* domain of biology. Once a *structural* whole, a body, is built, how is it *maintained* and how is its *activity* coordinated in time? How does such a body constructed of many parts *move* and *behave* as *one*, *coherent* unit? Can the presence of a SELFO in nearly all living systems help answer these questions?

### (a) Emergence of SELFOs in biological systems

To begin, we must consider what physics teaches us about the collective behaviour of non-living systems in which many individual subunits at a lower level of scale (e.g. individual H_2_O molecules) can give rise to various emergent properties at a higher level of scale (e.g. at the population level of many H_2_O molecules). There are three basic emergent phenomena non-living systems exhibit: total order (a solid in the case of water), total disorder (gas) or something in between (liquid) [92]. Unlike non-living systems, which tend toward equilibrium and can be found in any of these collective states, biological systems are generally considered to be self-organizing complex dynamical systems that tend to maintain themselves in the ‘somewhere in between’ category near the ‘edge of chaos’ where the system exhibits the most flexibility—not too ordered or rigid and not too disordered or chaotic [93,94]. Two main advantages of living on the ‘edge of chaos’ have been proposed: greater information flow through the system, and greater within-system flexibility of pattern formation and dissipation [95].

Given a vast potential state space, how biological systems maintain themselves within a critically narrow band of operation remains one of the major unanswered questions in biology. However, top-down feedback from higher levels of scale (e.g. organism) to subunits at lower levels (e.g. organs, cells in multicellular aggregates, proteins) is believed to play an important role [96–98]. Is it possible that SELFOs provide biological systems with electrical top-down feedback to maintain them in this dynamic, habitable state space? If so, how might they emerge from the lower-level subunits? Although the oscillations themselves have a similar character (figures 2 and 3), it is entirely likely they are generated by different mechanisms in different kinds of biological systems. We will now look at some possibilities in single-celled organisms, non-neural organisms and organisms with nervous systems.

**Figure 4.**
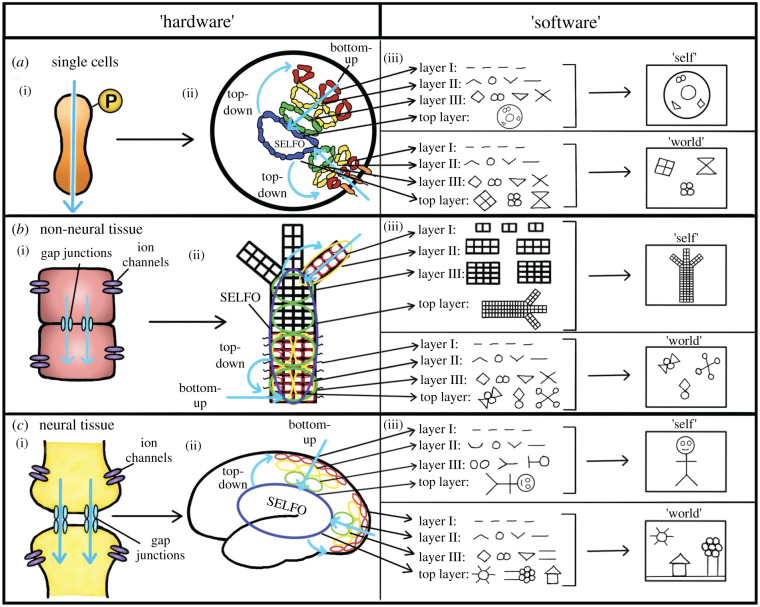
Electrical signalling throughout life. (*a*) Electrical signalling in single cells in which the ‘hardware’ may consist of (i) proteins that pass current (blue arrow) depending on their configuration, which can be altered with protein modifications (such as phosphorylation as shown). These ‘hardware’ pieces can be arranged in different networks within cells (ii) to form electrical circuits encoding information about the internal (top right circuit) and external (bottom right circuit) states, which can feed up to the spontaneous electrical low-frequency oscillator (SELFO) at the top level to be integrated to produce abstract representations (i.e. ‘software’) (iii) of both the cell's internal state (i.e. ‘self’ model) and external environment (i.e. ‘world’ model). The top-level SELFO can then feed back down to coordinate and update the lower-level components. (*b*) The same general architecture applies at the next level of scale in non-neural tissue where the ‘hardware’ becomes (i) non-neural cells that can be connected via gap junctions to allow the passage of ions intercellularly (blue arrows) while ion channels function to conduct ions intra- or extracellularly. These ‘hardware’ pieces can be arranged in different configurations within non-neural tissue to form electrical circuits encoding information about the internal (top right circuit) and external (bottom right circuit) states, which can feed up to the SELFO at the top level to be integrated to produce abstract representations (i.e. ‘software’) (iii) of both the organism's internal state (i.e. ‘self’ model) and external environment (i.e. ‘world’ model). The top-level SELFO can then feed back down to coordinate and update the lower-level components. (*c*) In organisms with nervous systems, the ‘hardware’ is upgraded to neurons (i) that, in addition to chemical synapses, can be connected via gap junctions to allow the passage of ions intercellularly (blue arrows) while ion channels function to conduct ions intra- or extracellularly. These ‘hardware’ pieces can be arranged in different configurations within neural tissue to form faster and more complex electrical circuits encoding information about the internal (top right circuit) and external (bottom right circuit) states, which can similarly feed up to the SELFO at the top level to be integrated to produce abstract representations (i.e. ‘software’) (iii) of both the organism's internal state (i.e. ‘self’ model) and external environment (i.e. ‘world’ model). As in the other cases, the top-level SELFO can feed back down to coordinate and update the lower-level components.

As already noted, despite conventional thinking that neuronal cells are unique in their ability to conduct electrical signals, many non-neural cells, from bacteria to various human cells, exhibit electrical activity in the form of subthreshold membrane potential oscillations and neuron-like action potentials (figure 3) [99–101]. These activities are generally thought to arise from the passage of ions through membrane ion channels. However, recent work suggests proteins, rather than being electrical insulators (as long thought), may conduct significant current depending on their conformation [102]. Using a scanning tunnelling microscope, researchers demonstrated that six randomly selected proteins previously assumed to be electrically inert all efficiently conducted current when bound to their cognate ligands in their native aqueous environment [102]. These findings challenge the view of proteins as primarily engaged in building cellular structures, catalysing chemical reactions, and transducing inter- and intracellular signals via post-translational modification [103]. Instead, these results suggest that, rather than protein modifications *being* the signal, they may serve to *affect* the ‘real’ electrical signal by allowing or prohibiting current flow through proteins by changing their configuration. Thus, proteins may act as subcellular electrical ‘hardware’ (figure 4*a*) serving as both ‘wires’ and ‘transistors’ that are ‘opened’ and ‘closed’ based on various protein modifications, which affect their ability to conduct current.

This new work supports an old idea originally proposed by Albert Szent-Györgyi in 1941 [104]: that proteins with highly regular structure might act as electron semiconductors within cells, similar to ‘non-living’ materials like crystals. This theory never took hold despite significant supporting evidence, including from Szent-Györgi himself in 1980 who demonstrated electronic conduction in a variety of dry proteins (e.g. casein, BSA, collagen, lysozyme)—conductivity that was similarly altered based on protein conformational changes due to both chemical and electrical modifications [105]. In parallel, Michael Berry put forth an ‘electrochemical model of metabolism’ in 1981 where he argued that cellular metabolic pathways (e.g. glycolysis, gluconeogenesis) can only be explained in terms of *both* chemical *and* electrical flows in which the flow of electrons and protons *through* proteins is critical for driving chemical reactions, not just within membranes, but, likely, throughout the entire cell [106–108]. Berry likened biological cells to ‘micro-electrode arrays' composed of two material phases: the ‘solid-state phase’ (i.e. the highly ordered ‘microtrabecular lattice’ made up of cytoplasmic proteins and organelles that can pass current), and the surrounding ‘bulk aqueous phase’ (i.e. the ‘electrolyte’, which can supply current). On this view, the ‘microtrabecular lattice' is seen as a ‘protoneural network’ in which electric current is passed within and between protein networks and organelles, which drives chemical reactions (see [106–108] for a full discussion of this complex topic). While this model remains to be fully verified (see [109] for a recent review calling for more research in this direction), the recent demonstration of electronic conduction within proteins in their native aqueous environments lends it further support [102].

Altogether, this work suggests the electrical properties of cells may be highly complex and dynamic with proteins binding together to form electrical circuits that are embedded in a changing electrical environment driven by ion flows within the ‘aqueous phase’ of the cytoplasm [106–108]. Given the alterations in protein conductivity observed with different chemical and electrical modifications [102,105,108], conduction through protein ‘wires’ would be expected to be highly dynamic and responsive to the surrounding chemical and electrical milieu. Such an intricate electrical landscape may be sufficient to generate a complex electrical oscillation frequency structure within cells, similar to those found in mammalian brains (figure 4). If this proposal is remotely correct, a SELFO might emerge bottom-up on the intracellular level as a consequence of complex interactions of many proteins passing electric currents and ions moving within the cytoplasm. Such a SELFO could, in turn, feed back down to coordinate and constrain those same lower-level subunits.

In multicellular organisms without nervous systems, mounting evidence suggests that electrical communication between somatic cells (the electrical ‘hardware’ at this level of scale, figure 4*b*) occurs both *directly*, via ion flow through cell–cell gap junctions, and *indirectly,* via extracellular ion flow through ion channels [14,101]. As we have seen, intercellular electrical communication exists in bacterial biofilms, when potassium ions are pumped out of single bacterial cells, causing neighbouring cells to release extracellular potassium through their membrane potassium channels, thus propagating a long-range potassium wave that travels from the inside of the biofilm to the periphery [12,13]. The spontaneous emergence of such biofilm-wide low-frequency electrical oscillations has now been mathematically modelled and shown to arise from an intricate interplay of bacterial metabolic stress that is communicated long range via electrical signalling to coordinate the individual bacterial cell responses within the group [110]. Thus, from the start, it appears mechanisms were in place to allow low-frequency electrical oscillations to spontaneously arise from complex interactions between groups of non-neural cells, which then serve as a ‘top-down’ mechanism within the system to coordinate and constrain the lower-level components.

In addition to the long-range extracellular electrical potassium waves found in bacterial biofilms, a growing body of evidence shows both extracellular and intercellular electrical communication occurs between cells in a wide variety of non-neuronal organisms [14,101]. It is thought each cell type possesses a unique resting membrane potential, which, in most cases, may oscillate over time [101]. Thus, when coupling these non-neuronal cells together via cell–cell gap junctions these subthreshold membrane-potential oscillations can be transmitted from somatic cell to somatic cell, creating distinct electrical circuits throughout the organism depending on how the cells are connected (figure 4*b*) [111]. These body-wide subthreshold membrane potential circuits have been shown to play critical roles in both the development and maintenance of overall body structure and have been proposed as another potential ‘top-down’ mechanism organisms use to coordinate their many parts [14,112]. After tissue injury, for example, it appears the modulation of organism-wide electrical signals *precedes* changes in molecular signals, suggesting the faster electrical signals are likely coordinating and constraining the slower molecular components [113]. This work suggests organisms build and maintain their bodies via a continuous complex feedback loop between subcellular molecular components (e.g. ion channels and gap junctions) that affect electrical activity on a higher level of scale (e.g. the circuit level), which then feeds back down to affect both the transcription and behaviour of the molecular components [14]. Thus, in addition to the classical molecular gradients that have been well-established in developmental biology since the discovery of the ‘head organizer’ [27], there appears to be an electrical activity gradient that likely arises out of the lower-level molecular components that might serve to coordinate and constrain those same molecular components. Given the complex interactions of the many underlying subunits at both the subcellular and cellular scale in non-neural organisms, it may be that a SELFO generating neuron-like action potentials emerges out of these interactions to communicate information organism-wide in a faster manner, as has been observed in both plants [5,6] and fungi [7–9] (figure 3).

Finally, how might SELFOs be generated in organisms *with* nervous systems? Neurons have long been regarded as the most efficient electrical ‘hardware’ in biology, conducting current rapidly through their long, one-dimensional ‘tubes’ (i.e. axons) and connecting to form circuits via both gap junctions and chemical synapses (figure 4*c*). Using these parts, it may be, as Passano originally proposed for the cnidarian nervous system half a century ago, that the highly conserved oscillation frequency structure observed in all mammalian brains arises as a ‘hierarchy of pacemakers' [2,3]. Most neurons exhibit intrinsic pacemaker activity [114,115]. That is, when isolated in culture, neurons from many different nervous systems exhibit ongoing, spontaneous electrical oscillations of varying frequencies. According to the classic Huygens' clock experiment [116], if you connect two oscillators of similar frequency they will synchronize and start oscillating together. If many intrinsically oscillating single neurons are connected, it is plausible to conjecture they might spontaneously form groups of oscillators (i.e. ensembles), oscillating at the same frequency. In this way, nervous systems of all shapes and sizes may spontaneously self-assemble into higher-level structures (i.e. ensembles of various sizes oscillating at various frequencies) forming a ‘heirarchy of pacemakers' in which the biggest, slowest oscillator in the system might serve to coordinate and constrain all of the smaller, faster oscillators (figure 4*c*).

### (b) Function of SELFOs in biological systems

Having considered how SELFOs may emerge bottom-up via a variety of mechanisms within biological organisms, we will now explore what they might do in more detail. Here, I propose three potential functions of SELFOs within living systems: (i) maintaining them at or near their critical point, (ii) integrating all the lower-level electrical information in the system, and (iii) continually communicating that high-level ‘view’ back down to the constituent components to both coordinate and update them on the overall state of the system to generate coherent, adaptive behaviour. While a thorough analysis of these potential functions is beyond the scope of this article, each will be briefly examined below.

#### (i) SELFOs maintain biological systems near criticality

As mentioned, one job of SELFOs may be to maintain organisms at or near their ‘critical point’ in state space by constraining them ‘top-down’ via their slow-wave electrical oscillations to allow both optimal information flow through the system and optimal flexibility of pattern formation and dissipation [95]. How might these properties be advantageous for living systems? Take the example of single cells, which contain many subcellular components (e.g. proteins). Like a glass of water, there are three general configurations a cell might be in with respect to its constituent parts, as discussed above: total order (proteins stuck in an unchanging state), total disorder/chaos (proteins moving about at random), and ‘somewhere in between’ (proteins form ‘patterns’—bind to each other to form useful structures—for a certain period of time before those patterns dissipate) [94]. To be most adaptive to its environment, a cell would do best by maintaining itself in the ‘somewhere in between’ state where patterns formed by its proteins are maintained for just enough time for them to be useful, but not too long such that they end up in a fixed, non-adaptive state (with all proteins stuck in one configuration, i.e. cell death) [93].

This advantage also applies to non-neural and neural organisms and is best understood in terms of the human brain, which is thought to maintain itself near criticality [117]. Interestingly, evidence suggests it is the SELFO in the human brain, the DMN, that might maintain the system near its critical point as disruption of the DMN results in more ‘fluid’ brain states in which neural activity patterns are more disordered and chaotic, correlating with psychedelic and psychotic states [57,118,119]. Conversely, over-active DMN activity results in more ‘stuck’ brain states in which neural activity patterns are more ordered, correlating with rumination and anxious or depressed states [57,117,120–122]. A totally ordered brain would be one in which either all neurons are off (i.e. brain death) or all neurons are firing in synchrony (i.e. seizure)—neither of which is a very useful state for the organism. Thus, SELFOs might serve to maintain biological systems near their critical point to allow both optimal information flow and pattern formation that is not too ordered nor too disordered.

#### (ii) SELFOs as organism-wide information integrators

The second role SELFOs may play in living systems is as organism-wide electrical information integrators. As discussed, all biological systems are composed of many constantly changing parts that must continually cooperate to form a unified whole that can both maintain its structure (i.e. its body) and move it to generate coherent, adaptive behaviour. This implies some part of the system must have access, however indirectly, to all the information within the system. No single subunit (e.g. single molecule in a cell or single cell in an organism) can have access to all the information in the system—that is the wrong level of scale [123]. However, a SELFO generated by those lower-level components, thus operating at a higher level of scale, could, in principle, receive information about all the lower-level subunits by integrating all the bottom-up electrical information in the system, as reviewed in Section 4 above, and outlined in figure 4. As such, SELFOs may act as the ultimate integrators in biological systems, which integrate all the lower-level electrical information being sent ‘up’ in increasingly higher levels of abstract representations of both the internal state of the system and the external environment the system is encountering. In this way, the SELFO would ultimately receive and ‘view’ all of the highest-level abstract representations of both the organism (i.e. its ‘self’) and its environment (i.e. its ‘world’), thus forming one integrated ‘self’/’world’ model (figure 4).

This view suggests the SELFO may thus continuously receive bottom-up electrical information from the entire system which it then might integrate over a specific time window based on its frequency, before taking a ‘snapshot’ of the organism and its environment—much like a camera chip integrates photons over a specified exposure time before taking a picture. Interestingly, biological SELFOs do not appear to maintain consistent oscillation frequencies. Rather, they appear to change their frequencies in response to different stimuli [2,6,7], as discussed above in the case of fungi that increase the frequency of their SELFOs in response to sulfuric acid, water, malt extract and wood, and decrease their frequency when the wood stimulus is removed [9]. Thus, it may be that SELFOs in each biological system have a specific baseline frequency range, determined by each organism's unique makeup, in which organisms might maintain a certain mid-range ‘baseline’ SELFO frequency that can be altered in response to both internal and external input. For example, if the organism is at rest and everything is as expected both internally and externally, the SELFO may integrate over a longer period of time (i.e. wait longer between snapshots of its ‘self’ and its environment) and thus update its ‘self’/’world’ model less frequently as nothing much is changing. However, if the organism encounters something unexpected (e.g. a predator is nearby) the SELFO may integrate over a shorter period of time (i.e. take more frequent snapshots of its ‘self’ and its environment) to increase its temporal resolution and update its ‘self’/’world’ model more frequently as it experiences faster change.

Another potential consequence of adjusting the SELFO frequency based on internal and external input may be a simultaneous modulation of the system's position in state space such that lowering the SELFO frequency (i.e. increasing its integration time) may result in a more ‘fluid’ system when it is ‘resting’ in an expected state and increasing the SELFO frequency (i.e. decreasing its integration time) may result in a more ‘constricted’ or ‘rigid’ system when it is in an unexpected or ‘stressed’ state.

#### (iii) SELFOs as organism-wide synchronizers and communicators

In addition to receiving all the electrical information in the organism, a third function SELFOs might serve is to transmit such integrated, high-level information back to their lower-level parts via organism-wide, synchronous firing to coordinate and constrain them. Ideally, the same signal would reach each component simultaneously such that the SELFO could serve as a ‘master clock’ for the organism to coordinate all parts in time. If biological systems are poised at or near criticality, information flow through the system would be optimal, ensuring the SELFO can both receive and send system-wide information most rapidly [95]. Unlike typical machine clocks, however, which are precisely designed to maintain regular oscillations to ensure consistent timing devices [124], the SELFO ‘master clock’ found in biological organisms appears to be constantly altering its frequency based on internal and external input [2,6,7,9,10], as previously discussed. Hence, in addition to serving as a timing device, SELFOs/biological ‘master clocks’ may also transmit information about the state of the system by changing their frequencies (i.e. changing their clocking intervals). The SELFOs/biological ‘master clocks’ thus appear to be intrinsically adjustable oscillators (i.e. adjustable clocks), adjusted internally by their own continually changing components, in contrast to adjustable oscillators in machines, like radios, which must be adjusted externally [124].

Such an intrinsically adaptable SELFO/biological ‘master clock’ would allow the organism to adjust three main parameters on-the-fly simultaneously, as partially reviewed above: (i) its integration time (i.e. how long it ‘reads’ bottom-up information to get a ‘snapshot’ of its ‘self’ and its ‘world’), (ii) its position in state space (i.e. how ‘fluid’ or ‘rigid’ the system is), and (iii) how often it will update its lower-level components. As discussed above, if an organism is at rest and everything is as expected it might want not only to integrate and update its ‘self’/’world’ model less frequently, but also to update its lower-level components less frequently to conserve energy as firing action potentials is energetically expensive [47]. Alternatively, if the organism encounters something unexpected it might increase its firing rate not only to integrate and update its ‘self’/’world’ model more frequently, as above, but also to update its downstream components more often to alert them of potential internal or external changes to the system or its environment. Thus, the synchronous firing of the SELFO might not only provide top-down sub-component coordination to ensure organism unity, it might also communicate information about the overall state of the system via changes in its frequency—changes that are a result of ongoing bottom-up input from all of its constituent parts, thus making it a highly adaptable intrinsically adjustable oscillator/‘master clock’.

## 7. Conclusion

The picture sketched above is highly speculative. However, there is a real phenomenon to be explained—SELFOs, which are highly conserved in mammals, have been ‘discovered’ multiple times in Cnidaria and now in many other widely divergent phyla, including plants and single-celled organisms. To date, this activity has attracted little attention outside of human neuroimaging studies with poor spatial and temporal resolution.

As the only animal whose entire nervous system can currently be imaged simultaneously at single-cell resolution while behaving [1], *Hydra* will have an important role to play in this investigation. It remains unclear if the *Hydra* SELFO, its RP1 network active ‘at rest,’ is also involved in generating behaviour (elongation) as proposed in the recent work (figure 1) as there was not an obvious relationship between RP1 activity and elongation, causing the authors to speculate that rather than directly *generating* behaviour, RP1 may serve to integrate sensory information and *coordinate* behaviour [1]. This is an important distinction that can be tested in further high-resolution studies of the freshwater polyp in which RP1 activity (its frequency, amplitude and phase) can be precisely measured during different behaviours and during ‘rest’ to more definitively determine whether the *Hydra* SELFO is only involved in non-behaviour-generating processes (i.e. ‘rest’ and behaviour coordination) or also plays a role in direct behaviour-generation. In addition to its relationship with *Hydra* behaviour, the relationship of RP1 activity with the other neural networks can also be rigorously assessed to determine if it does, indeed, coordinate them, and, if so, precisely how. The most definitive experiment would be disruption of the *Hydra* SELFO by optogenetic, pharmacologic or physical means. Two major findings would be expected based on the above hypotheses regarding the potential role of SELFOs: (i) a more ‘disordered’ *Hydra* nervous system owing to the loss of ‘top-down’ feedback to keep the system at or near criticality, and (ii) less coordinated behaviour owing to the loss of organism-wide electrical information integration and communication required to maintain organism unity.

In addition to allowing loss-of-function experiments, *Hydra* is also uniquely suited to study the natural development and function of SELFOs as it reproduces asexually by budding [125]. This allows the study of how multiple bodies (i.e. buds and parents attached to each other) might function as one coherent organism while sharing the same synchronous SELFO (i.e. sharing the same electrical organism organizer) and subsequently start to function as multiple uncoordinated, individual organisms (while still physically attached) with the development of asynchronous, separate SELFOs (i.e. two separate electrical organism organizers). *Hydra* also possesses remarkable regenerative capacities [20], allowing myriad cutting and grafting experiments in which animals of all shapes and sizes can be generated with varying numbers of neurons and SELFOs to explore how coordinated versus uncoordinated activity might emerge in these structures (e.g. *Hydra* with multiple heads, multiple feet, no head, no foot, or any combination thereof). Not only does *Hydra* regenerate when cut, it also forms a new whole animal from totally dissociated single cells [126], allowing the study of the emergence of SELFOs and organism-wide coordination within a group of dissociated elements. Lastly, an adult *Hydra* is constantly rebuilding itself, turning over all of its parts every 20 days [127], which allows the study of how an organism maintains its body, its nervous system and SELFO, and coherent behaviour despite ever-changing components.

Since the discovery of *Hydra* over 300 years ago, this simple animal has taught us a great deal about biological systems—primarily, how organisms build their bodies using molecules. Now, *Hydra* is beginning to reveal the secrets of its nervous system, in which ‘cryptic’ SELFOs have been lying in wait since their initial discovery in the 1960s. It is no surprise such spontaneous neural activity was overlooked as there was no place for it within the dominant input–output paradigm inherited from Sherrington [45]. The recent unexpected discovery of a SELFO, the DMN, in the human brain, however, has required a substantial revision of such a ‘reflex’ model of nervous systems and reignited interest in endogenous neural activity. Since its discovery, the DMN has become increasingly linked to the ‘self’ in humans, potentially acting as a brain-wide integrator, but its precise function and mechanism remains obscure given the limitations of both imaging and manipulating human brains. Interestingly, the same kind of spontaneous electrical activity found in the human DMN appears to be highly conserved throughout life. The widespread presence of SELFOs suggests they may be playing an important role in organism-wide integration and communication in biological systems at all levels of scale and opens the door to their study in more experimentally tractable systems, such as *Hydra*. As throughout the history of biology, this basal animal is poised to once again teach us about another fundamental aspect of living systems: this time, how organisms create and maintain coherent, adaptive wholes using electricity. Insights gained in *Hydra*, as before, are likely to apply to biological systems at all levels of scale, from bacteria to humans, and have important implications for psychiatry, neurology and, potentially, tumorigenesis.

## Data accessibility

This article does not contain any additional data.
